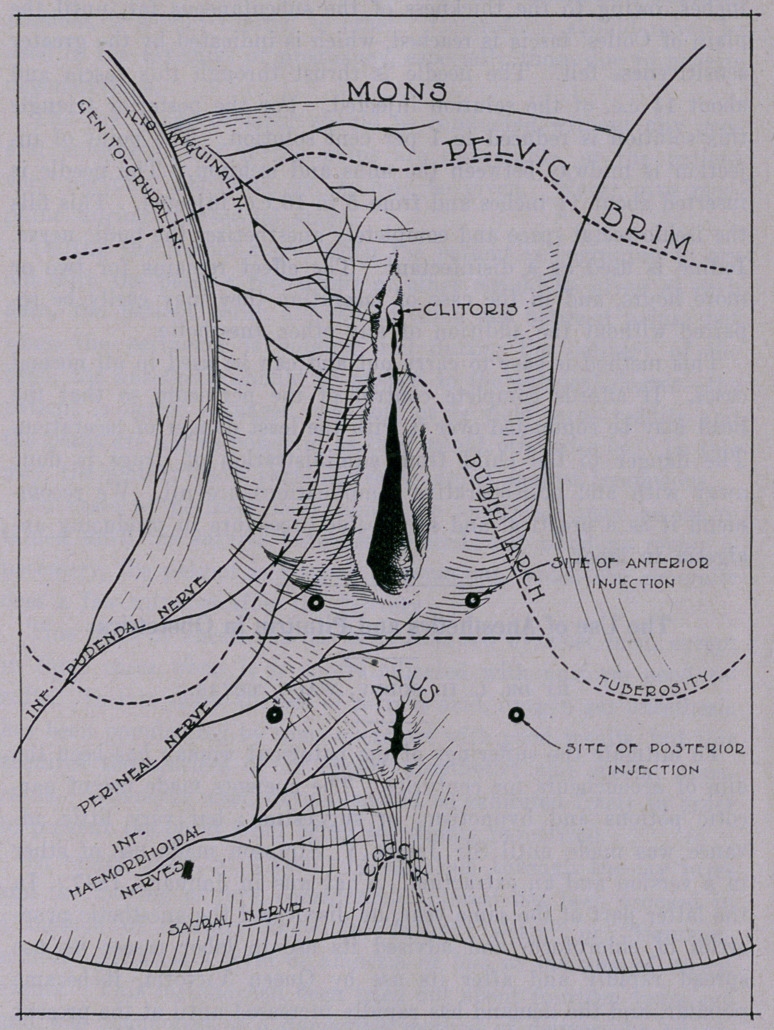# Anoci-Association Applied in Obstetrics

**Published:** 1917-05

**Authors:** Malone Duggan

**Affiliations:** San Antonio, Texas


### TEXAS MEDICAL JOURNAL

DR. F. E. DANIEL,
Founder.

MRS. F. E. DANIEL,
Publisher and Managing Editor.

PUBLISHED MONTHLY.
Subscription:
$1.00 a Year.

Established July, 1885.

VOL. XXXII No. 11
AUSTIN,
MAY, 1917.

The publisher is not responsible for views of contributors.

*"All of good the past hath had,*
*Remains to make our own time glad."*—WHITTIER.

### Original Articles.

### Anoci-Association Applied in Obstetrics.

BY DR. MALONE DUGGAN, SAN ANTONIO, TEXAS.

My object in presenting this subject to you is to call attention to this form of anesthesia in obstetrics, as I believe it offers the most practical solution of this difficult question. I had hoped to have had several case reports to make, but, owing to unforeseen circumstances, I have but one. This, however, will serve the purpose of introducing a discussion.

Mrs. S., age 28 years, multipara, L. O. A. First child born three years ago; second child born February 16. No previous sickness, except a chronic catarrh of the nose and stomach. Has a neurotic heredity, though she is not nervous. First pregnancy was a ten-months' gestation, otherwise it was normal. Suffered intensely from hyperuresis during the first three months of this pregnancy, and, during the last two months, she was compelled to keep her bed, owing to intense pain in the pelvis, brought on when standing on her feet. Because of this condition, together with the history of the previous labor, having gone over time, we decided to induce labor at the completion of the full period of gestation.

This was done by the insertion of two sterile bougies into the womb, passed up to the fundus. Natural first-stage labor pains were thus induced in the course of a few hours, and continued in a very normal manner. In eight hours the second stage of labor was reached, which was normal, except that the pains lacked propulsive force. Gas-oxygen anesthesia was begun in the latter part of the first stage and continued through the entire second stage except at the very last. This simply consisted of a few whiffs of the gas just at the beginning of each pain and was withdrawn during the intervals. The result of this was to calm her fears, and no doubt it relieved, to some extent, her pains. As the head engaged the outlet and the perineum began to bulge, I injected 1 c.c. of a 2 per cent novocaine solution into each anterior triangle, and 5 c.c. of a 1 per cent solution into each of the posterior triangles. This being a much less quantity than advocated by King, I was not surprised at not getting the full anesthetic result as expected. But I desired to see to what extent anesthesia could be produced, so began with the smaller dose. As far as I could determine, the effect was not marked, and I resorted to ether during the last expulsive pains. However, I could see that the principle was rational and I believe if pressed to complete infiltration of the triangles it would succeed admirably.

The argument according to Hoag, S. G. & O., 11-16, is that if nerve-blocking will induce relaxation in abdominal section under gas-oxygen anesthesia, why would it not do the same in relaxing the perineum? He argues that the trouble with gas-oxygen anesthesia alone in obstetrics was its failure to completely relax the perineum at the crucial moment, and suggest either the use of ether or nerve-blocking to bring about this effect. In his hands, preliminary small doses of scopolamin during the first stage, nitrous-oxide oxygen during the pains and nerve-blocking, when the perineum begins to bulge, give ideal results. His method of nerve-blocking is to infiltrate the tissues around the rim of the vagina with a large injection of novocaine solution, followed with a 1 per cent quinine and urea solution. The success of the procedure depends upon the thoroughness with which the infiltration is accomplished.

But this is too complicated a method to carry out very well outside of a hospital. King, of Denver, Colorado, in the same journal, proposes a method of nerve-blocking that eliminates gas and ether anesthesia altogether. This author bases his procedure on the principle that the stretching of the cervix does not cause much pain; that the severe pain of labor is due to the stretching of the soft parts, especially the rupture of Colles' fascia. For convenience, he divides the perineum into a posterior and anterior triangle by a straight line connecting the ischia passing midway between the anus and vaginal outlet. In the anterior triangle we have the inferior pudendal, genito-crusal and ilio-inguinal nerves. In the posterior triangle are some of the branches of the sensory nerves, Colles' fascia, ischio-rectal fascia and the triangle ligament. To completely infiltrate these triangles on both sides, according to King, will control the pain caused by the stretching and tearing of the soft parts. It is only necessary to inject the anterior triangles in primipara, but both on each side should be injected in all cases where the perineum has been previously torn.

Dr. King's technique is to provide a 2 per cent solution of novocaine with the addition of 3 drops of 1-1000 adrenal chloride to the c.c. For the anterior triangles he selects a point ¾ inch from the lower margin of the vagina and an equal distance from the ramus of the ischium. A needle is inserted from ¾ to 1½ inches, owing to the thickness of the subcutaneous fat, until the plain of Colles' fascia is reached, which is indicated by the greater sensitiveness felt. The needle is thrust through this fascia and about 1½ c.c. of the solution injected. For the posterior triangle this solution is reduced to 1 per cent solution. The point of injection is midway between the anus and ischium. The needle is inserted about 1½ inches and from 5 to 10 c.c. injected. This fills the ischio-rectal space and completely anesthetizes the pudic nerve. Iodine is used as a disinfectant. The effect remains for two or more hours, and in the case of laceration they may easily be repaired without the addition of any other anesthetic.

This method is easy to carry out and can be used in all normal cases. It affords complete control of the perineum so that the head may be conducted over it with the least danger of laceration. The danger to the child from administration of drugs is done away with and post-operative complications are nil. We recommend it as a practical and serviceable procedure in producing analgesia in labor.

## Figures and Tables

**Figure f1:**